# Hematological ratios as an indicator of severity in alopecia areata: A retrospective nationwide study

**DOI:** 10.1371/journal.pone.0314600

**Published:** 2024-12-02

**Authors:** Nicolas Andre, Sarah Weissmann, Bracha Cohen, Chaya Bracha Gordon, Majd Nassar, Inbal Kestenbom, Inbal Golan-Tripto, Amir Horev

**Affiliations:** 1 Faculty of Health Sciences, Ben Gurion University of the Negev, Beer-Sheva, Israel; 2 Clinical Research Unit, Soroka University Medical Center, Beer Sheva, Israel; 3 Pediatric Endocrinology Unit, Soroka University Medical Center, Beer Sheva, Israel; 4 Pediatric Emergency Department, Soroka University Medical Center, Beer Sheva, Israel; 5 Pediatric Pulmonary Unit, Soroka University Medical Center, Beer Sheva, Israel; 6 Pediatric Dermatology Service, Soroka University Medical Center, Beer-Sheva, Israel; Universiti Malaysia Sabah, MALAYSIA

## Abstract

**Background:**

Alopecia Areata (AA) is an autoimmune condition where the activation of Th1, Th2, and Th17 responses is known to stimulate other white blood cells, potentially affecting hematopoietic lineages. However, previous studies on AA have found no utility in hematological ratios. Our goals were to compare neutrophils-to-lymphocytes ratio (NLR), platelets-to-lymphocytes ratio (PLR), eosinophils-to-lymphocytes ratio (ELR), eosinophils-to-neutrophils ratio (ENR), and eosinophils-to-monocytes ratio (EMR) between patients with AA and controls, as well as between mild and moderate-severe AA cases.

**Methods and findings:**

We performed a retrospective, population-based cohort study involving adult patients enrolled in the largest national health maintenance organization in Israel. The study comprised 147,020 AA patients and 141,598 healthy controls. AA patients exhibited a higher likelihood of elevated NLR and ELR compared to controls. Upon further classification based on severity, moderate-severe AA patients displayed higher values of NLR, PLR, ELR, and EMR compared to mild AA individuals OR = 1.11 [1.09–1.1], P<0.001; OR = 1.09 [1.05–1.13], P<0.001; OR = 2.06 [1.67–2.53], P<0.001; OR = 1.07 [1.03–1.07)], P<0.001, respectively). Similar trends were observed 12 to 18 months before diagnosis.

**Conclusion:**

Our results not only deviate from the current literature but also offer a cost-effective, accessible, and efficient tool for enhanced disease prediction and management.

## Introduction

Alopecia Areata (AA), a non-scarring form of hair loss, is characterized by the immune system targeting hair follicles (HF) in the anagen phase, leading to the loss of HF immune privilege [1]. It affects up to 2% of the general population [2]. Besides hair loss, this condition can lead to severe consequences, imposing a considerable psychological burden on patients, often resulting in heightened levels of anxiety and depression [3].

Previous research on AA has gained better insight into the immune cells and cytokines involved in its pathophysiology [4]. Studies have given evidence that the breakdown of the HF immune privilege following triggering events, such as viral infections, initiates an autoimmune response. This was shown to involve a Th1 response whereby autoreactive cytotoxic CD8+NKG2D+ T cells primarily target exposed HF autoantigens [5]. Furthermore, recent findings suggest that both a Th2 immune response and a Th17-mediated response may play roles in targeting and destroying bulge stem cells and hair bulb matrix stem cells, further contributing to severe hair loss [6, 7].

As these immune responses are recognized to activate other white blood cells, alterations in hematopoietic lineages within the bloodstream can be anticipated and may serve as valuable indicators for assessing and predicting the prognosis of this condition. This has been previously investigated in other autoimmune and inflammatory diseases, such as vitiligo [8] and psoriasis [9], by exploring various ratios such as the platelet to lymphocyte ratio (PLR), eosinophil to lymphocyte ratio (ELR), eosinophil to neutrophil ration (ENR), eosinophil to monocyte ratios (EMR) and neutrophil to lymphocyte ratio (NLR).

Previous investigations on hematological ratios in AA have yielded inconclusive findings. Dere et al. concluded that parameters like PLR, NLR, and mean platelet values were not suitable indicators for demonstrating inflammatory response in AA [10]. Similar conclusions were drawn by İslamoğlu et al. [11] However, these findings could be attributed to small sample sizes as well as a limited use of other ratios, suggesting that these studies may not accurately reflect the overall significance of hematological ratios in AA.

In our study, we aimed to evaluate and contrast NLR, PLR, ELR, ENR, and EMR ratios across patients with AA and controls, and patients diagnosed with severe versus non-severe AA, within a larger cohort. This investigation seeks to enhance our understanding of the clinical significance of these ratios in the assessment, prognosis, and/or management of AA.

## Materials and methods

We conducted a retrospective, population-based cohort study on adults patients (18 years old and above) diagnosed with AA, within the national database of Clalit HMO (Health Maintenance Organization) or treated at Soroka University Medical Center (SUMC), from 2005 to 2020. Clalit HMO is Israel’s largest public healthcare provider and serves over 4.6 million people. SUMC is a 1,200-bed university-affiliated referral center in Southern Israel that functions as a tertiary hospital for over 1 million people. Approval from Soroka Medical Center’s institutional review board was obtained, with an exemption from written consent requirements, prior to the study which complied with the 1964, 1975 and 2013 revisions of the Helsinki Declaration (approval number 0434-15-SOR).

Our study included adults diagnosed with AA who were either Clalit HMO insured or received care at SUMC. Demographic and medical data were sourced from primary care physicians, dermatologists, and SUMC’s Admission-Discharge-Transfer (ATD) hospital system. Data extraction was done via Clalit’s data-sharing platform, powered by MDClone software, on September 22, 2022. AA diagnoses were established by primary physicians or dermatologists. Authors did not have access to information that could identify individual participants during or after data collection. The following International Classification of Diseases– 10 were used to gather our data: ALOPECIA AREATA L63.9 and OPHIASIS L63.2 [12]. Exclusions comprised patients with recent infections, malignancies, recent surgeries, or lacking blood tests within 30 days preceding AA diagnosis. We randomly matched our cohort of AA patients with healthy controls by gender and birth year using R Studio. Controls were individuals without a diagnosis of AA insured by Clalit HMO. We then obtained blood count data from the healthy controls within a year of their matched patient’s blood test. Finally, we excluded all controls with infection, malignancy or surgery within 30 days of their blood test.

Our study comprised a total of 147,020 participants diagnosed with AA. After implementing all exclusion criteria, we successfully identified 141,598 matched controls. Calculations of eosinophil/lymphocyte ratio (ELR), eosinophil/monocyte ratio (EMR), and eosinophil/neutrophil ratio (ENR) were performed using complete blood counts within 30 days of initial AA diagnosis as well as 12–18 months before diagnosis of AA. Patients were classified as having moderate-severe AA if they were prescribed systemic AA-related medications or received intralesional injections, while others were considered mild.

Age, ELR, ENR and EMR were normally and the Student’s t-test was used. For gender, SES, smoking status, and ethnicity we applied the Chi squared test. Multivariable logistic regression was also performed, including blood ratio, age, sex, ethnicity, socioeconomic status, and smoking status (with available data). A significance level of P < 0.05 and 95% confidence intervals was applied. The receiver operating characteristic (ROC) curve was used to assess the predictive value of hematologic ratios on severity. We obtained optimal cut-off values using the Youden’s Index. Statistical analyses were conducted using R software (version 4.0.2).

## Results

A significantly higher proportion of patients were female compared to males, constituting 80.9% (n = 118,969, P<0.001). The majority of patients identified as Jewish, accounting for 75.7% (n = 111,336). 12.1% of the patients (n = 17,783) had moderate-severe AA (Table 1).

**Table 1:** Clinical and demographic characteristics of patients with AA compared to controls.

Characteristic	Patients with AA	Controls
N	147,020	141,598
Gender (%)		
Male	28,050 (19.1)	24,819 (17.5)
Female	118,969 (80.9)	116,779 (82.5)
Ethnicity (%)		
Arab	26,996 (18.4)	28513 (20.1)
Jewish	111,336 (75.7)	103,323 (72.9)
Other	8,688 (5.9)	9,762 (6.8)
Severity	17783 (12.1)	

After categorizing our data into mild and moderate-severe cases, it was determined that 87.9% of our patients fell into the mild category (n = 129,237), while 12.1% were classified as moderate-severe (n = 17,783, detailed breakdown can be found in Tables 2 and 3).

**Table 2:** Hematologic ratios of patients with AA, stratified by severity.

Characteristic	Test statistic	Degrees of Freedom	Mild	Moderate-Severe	P-value	95% confidence interval
N			158,990	19895		
Age^1^	-59.24	21971	39.09 (17.11)	47.96 (18.92)	**<0.001**	**-9.56, -8.57**
Gender (N (%))^1^	3.48	2			0.175	
Female			104,489 (80.9)	14,480 (81.4)		
Male			24,747 (19.1)	3,303 (18.6)		
Ethnicity (N (%))^1^	109.5	2			**<0.001**	
Jewish			98,005 (75.8)	13,331 (75.0)		
Arab			23,313 (18.0)	3,683 (20.7)		
Other			7,919 (6.2)	769 (4.3)		
NLR (mean (SD))^1^	-18.56	20815	1.83 (0.91)	2.00 (1.18)	**<0.001**	**-0.19, -0.15**
PLR (mean (SD)) ^1^	-8.39	21657	122.95 (44.63)	126.34 (51.23)	**<0.001**	**-4.18, -2.59**
ELR (mean (SD)) ^1^	-10.09	20948	0.09 (0.07)	0.10 (0.09)	**<0.001**	**-0.008, -0.005**
ENR (mean (SD)) ^1^	-1.58	7264	0.06 (0.05)	0.06 (0.07)	0.563	-0.003, 0.0003
EMR (mean (SD)) ^1^	-2.80	21152	0.50 (0.39)	0.51 (0.48)	**0.001**	**-0.018, -0.003**
NLR 12–18 months before (mean (SD)) ^1^	-3.70	13144	2.13 (1.52)	2.20 (1.51)	**<0.001**	**-0.095, -0.029**
PLR 12–18 months before (mean (SD)) ^1^	-4.53	12365	127.65 (51.33)	130.48 (56.79)	**<0.001**	**-4.05, -1.60**
ELR 12–18 months before (mean (SD)) ^1^	-7.64	12134	0.09 (0.07)	0.10 (0.08)	**<0.001**	**-0.008, -0.005**
ENR 12–18 months before (mean (SD)) ^1^	-4.05	12168	0.05 (0.05)	0.05 (0.0)	0.060	-0.003, -0.001
EMR 12–18 months before (mean (SD)) ^1^	-3.07	12613	0.48 (0.39)	0.49 (0.42)	**0.001**	**-0.02, -0.005**

^1^ Welch Two Sample T-test

^2^ Pearson’s Chi-squared test

**Table 3:** Comparison of hematological ratios of moderate-severe patients with mild patients with AA.

Hematological ratios*		Mild AA	Moderate to Severe AA	Total	OR** (95%CI)	p-value
NLR	Low	63668	7942	71610	1.11 (1.09–1.13)	**<0.001**
	High	65569	9841	75410		
PLR	Low	64841	8670	73511	1.09 (1.05–1.13)	**<0.001**
	High	64396	9113	73509		
ELR	Low	65603	8338	73941	2.06 (1.67–2.53)	**<0.001**
	High	63634	9445	73079		
EMR	Low	64705	8805	73510	1.07 (1.03–1.07)	**<0.001**
	High	64532	8978	73510		

* Dichotomized into low and high based on median.

** Obtained through logistic regression analysis

***P-value <0.05 was significant

Gender appeared to have no significant impact on the severity of AA (P = 0.175). Patients identified as moderate-severe tended to receive their diagnosis later in life compared to those classified as mild (47.96 years vs 39.09 years, P<0.001). Moreover, individuals with moderate-severe AA demonstrated higher NLR, PLR, ELR, and EMR values compared to those with mild AA (Table 2).

Furthermore, upon conducting a regression analysis, we found that moderate-severe AA patients exhibited a higher likelihood of having elevated levels of these same ratios compared to mild patients (Table 3). This pattern was consistent even when examining complete blood counts 12 to 18 months preceding the diagnosis of AA (Table 3).

AA patients were more likely to have an elevated NLR and ELR compared to matched controls (OR = 1.02 [1.01–1.03], P<0.001; OR = 1.21 [1.05–1.39], P = 0.007 respectively), they exhibited comparable PLR, ENR and EMR (OR = 1.01 [0.99–1.03], P = 0.5; OR = 0.98 [0.78–1.22], P = 0.8; OR = 1.02 [0.99–1.05], P = 0.2).

The Youden indices in Table 4 identified the best cut-off values for predicting severe AA. At the time of diagnosis, optimal cut-off values were 1.87 for NLR, 126.36 for PLR, 0.09 for ELR, 0.59 for EMR, and 0.09 for ENR, with AUCs of 0.54, 0.51, 0.52, 0.50, and 0.52, respectively. For the period 12–18 months before diagnosis, the best cut-off values were 1.85 for NLR, 141.20 for PLR, 0.08 for ELR, 0.50 for EMR, and 0.08 for ENR, with corresponding AUCs of 0.52, 0.51, 0.53, 0.51, and 0.53. These values were used to predict moderate-to-severe AA.

**Table 4:** Youden Indexes for moderate-to-severe alopecia areata.

Characteristic	Threshold			Specificity	Sensitivity		AUCs^1^
**NLR**		**1.87**	**0.62**			**0.44**	**0.54**
**PLR**		**126.36**	**0.69**			**0.33**	**0.51**
**ELR**		**0.09**	**0.62**			**0.43**	**0.52**
EMR		0.59	0.71			0.29	0.50
**ENR**		**0.09**	**0.62**			**0.43**	**0.52**
**NLR 12–18 months before**		**1.85**	**0.52**			**0.52**	**0.52**
**PLR 12–18 months before**		**141.20**	**0.69**			**0.33**	**0.51**
**ELR 12–18 months before**		**0.08**	**0.57**			**0.48**	**0.53**
**EMR 12–18 months before**		**0.50**	**0.69**			**0.33**	**0.51**
**ENR 12–18 months before**		**0.08**	**0.57**			**0.48**	**0.53**

^1^ AUC = Area Under the Curve

## Discussion

Our study represents the first of its kind in analyzing a comprehensive array of hematological ratios within such a large cohort. While the NLR was statistically higher in AA patients, we believe that the ELR was a more robust ratio for distinguishing AA patients from matched controls, as its odds ratio was greater than that of the NLR.This trend was largely consistent when assessing ratios in individuals 12 to 18 months before AA diagnosis. Despite females constituting a significant majority, gender did not appear to significantly influence AA severity. However, age did, with moderate-severe patients being diagnosed later in life than mild patients. Furthermore, individuals with moderate-severe AA displayed elevated NLR, PLR, ELR, and EMR values compared to those with mild AA, a trend supported by our regression analysis. This pattern persisted even when examining complete blood counts 12 to 18 months prior to AA diagnosis.

Our findings reveal that patients with AA exhibited higher NLR and ELR levels compared to their matched controls. Eosinophils have been implicated in several autoimmune conditions, including autoimmune myocarditis and inflammatory bowel diseases [13], and have also been reported in certain autoimmune dermatological conditions such as vitiligo [8]. Magen et al. demonstrated a high prevalence of food allergy and chronic spontaneous urticaria in patients with AA [14], further suggesting an increase in eosinophils in AA. Similarly, neutrophils are early responders in the course of tissue damage that have been implicated in the pathogenesis of immune diseases such as rheumatoid arthritis and systemic lupus erythematous [15].

Our findings are compelling as they appear to favor almost all of the studied ratios for assessing the severity of AA. NLR has emerged as a marker of diseases in biomedical research and is widely recognized as an indicator of immune system homeostasis [16]. Along with NLR, PLR was demonstrated to be elevated in atopic dermatitis, as shown in a study by Jiang et al. [17] Similarly, ELR and EMR are well-established markers of inflammation, with studies indicating significant elevation in patients with various allergic and inflammatory conditions such as urticaria [18], acute ischemic stroke [19], allergic rhinitis [20] and Kawasaki disease [21]. Çekici et al. confirmed in their observational study that high NLR and ELR were useful indicators of systemic inflammation activity [22]. Our study not only aligns with these findings but also contributes a new perspective by exploring their relevance in the context of autoimmunity. This further corroborates the observations of Weissmann et al., who noted the utility of ELR in the assessment and prognosis in severe vitiligo patients [8] as well as the NLR in severe psoriasis [23]. Though we found relationships between AA and these biomarkers, the predictive accuracy of NLR, PLR, ELR, EMR and ENR are relatively low, highlighting the need for a multimodal approach to prediction that includes clinical as well as laboratory factors.

However, our findings extend beyond the assessment of NLR, PLR, ELR and EMR elevation solely in patients diagnosed with moderate-severe AA; they were also elevated 12 to 18 months before exacerbation of the disease. This is particularly remarkable, as these markers could serve not only as indicators of severity but also as predictive factors for severity at least one year before the exacerbation of the condition. Therefore, it is conceivable that these two hematological factors could serve as valuable tools in determining disease severity and guiding appropriate management strategies. These findings stand in stark contrast to the prevailing literature on AA and hematological ratios, which suggests that such ratios are not effective as inflammatory markers for AA [10, 11]. They demonstrate that not only NLR, PLR, ELR and EMR are effective in this regard, but also offer a cost-effective means to predict severity more than a year before the exacerbation of the disease.

Our findings reveal an intriguing relationship between the onset age of AA and its severity. Specifically, we observe that severe cases often manifest later in life. This observation is noteworthy given that early onset age is usually considered a prognostic indicator, as it has been found in certain autoimmune diseases like systemic lupus erythematosus [24] or psoriasis [25]. Conversely, certain dermatological conditions, such as hidradenitis suppurativa, have been associated with increased severity when onset occurs later in life [26]. Our study aligns with these observations within the context of AA and offers fresh perspectives on tailoring treatment approaches based on patient age. Further research is warranted to deepen our understanding of how age influences the severity of AA.

Our study provides valuable new clinical insights into the management and severity of AA. It is particularly intriguing to consider that these clinical implementations does not require additional laboratory tests, but can be derived from ratios using existing laboratory results. Although our research identifies the elevation of hematological ratios, particularly ELR, in relation to AA severity, more studies are required to establish a clear threshold for determining severe and mild disease. We used the Youden Index to identify optimal cut-off values that balance sensitivity and specificity, yet the AUC values remained below 0.6, a level generally regarded as minimally acceptable for diagnostic use. The limited sensitivity and specificity of these blood markers may stem from the complex pathogenesis of AA, involving both innate and adaptive immune responses, which suggests that single inflammatory ratios may not fully capture the nuances necessary for accurate severity prediction. Further prospective research is required to obtain robust statistical data on the specificity and sensitivity of each ratio in both severe and mild cases, in order to better determine which ratios are most effective for clinical use. Regardless, we believe that the association we have identified could be beneficial for future research on the immunogenesis of AA, ultimately contributing to the development of treatments for the disease.

Our study possesses several strengths. Firstly, the inclusion of a large number of patients, totaling more than 147,000 AA patients, enables us to place significant confidence in the results obtained and significantly enhances the robustness of our study. Moreover, our patient selection was not confined to a single region but encompassed an entire country, greatly enhancing the generalizability of our findings. However, our study also has its limitations. Firstly, the retrospective nature of this investigation introduces potential biases related to data collection and the assessment of disease severity. This is particularly true for comorbidities or treatment histories, which could have affected white blood cell counts in various ways and ultimately influenced our results, despite the large cohort size and our exclusion criteria. Additionally, the diagnosis was established by a dermatologist and primary care physicians, potentially leading to the inclusion of a small number of patients with other types of alopecia.

In conclusion, our retrospective examination of hematological ratios in assessing AA, involving a considerable patient cohort, revealed that elevated NLR, PLR, ELR and EMR serve as four different ratios useful for evaluating AA severity. Furthermore, we observed this pattern 12 to 18 months before disease exacerbation. Although further studies are necessary to refine the sensitivity and specificity of these markers, especially in conjunction with other blood markers, these findings not only diverge from existing literature but also provide a cost-effective, available and efficient tool for improved disease prediction and management.
